# Does Perfectionism Lead to Well-Being? The Role of Flow and Personality Traits

**DOI:** 10.5964/ejop.1987

**Published:** 2021-05-31

**Authors:** Tamar Kamushadze, Khatuna Martskvishvili, Maia Mestvirishvili, Mariami Odilavadze

**Affiliations:** a Department of Psychology, Ivane Javakhishvili Tbilisi State University, Tbilisi, Georgia; Connection Lab, San Francisco, CA, USA

**Keywords:** adaptive and maladaptive perfectionism, psychological well-being, flow, HEXACO

## Abstract

Perfectionism is a personality trait that plays an important role in understanding human behavior and functioning. There has been a focus on the negative aspects and outcomes of perfectionism, and less is known about whether and how perfectionism relates to adaptive characteristics of personality and normal functioning. We investigated associations between different aspects of perfectionism and psychological well-being in two studies by determining the role of dispositional flow and personality traits in this relationship. In Study 1, participants completed questionnaires for perfectionism, psychological well-being and flow. In Study 2, personality traits from the HEXACO model of personality were additionally measured. We found that psychological well-being had a positive correlation with conscientious perfectionism and a negative correlation with self-evaluative perfectionism. Flow mediates the relationship between conscientious perfectionism and psychological well-being. There was no correlation between self-evaluative perfectionism and dispositional flow. After controlling for relevant personality traits, dispositional flow remains the mediator between conscientious perfectionism and psychological well-being, but the relation becomes negative. Implications for the understanding of how different components of perfectionism are related to psychological well-being and how flow experience contributes to this relationship are discussed.

Perfectionism has been described as an individual difference variable characterized by striving for excellence and motivation to pursue perfection. It involves setting excessively high standards and critically evaluating one’s behavior (Hewitt & Flett, 1991; Stoeber & Otto, 2006). As perfectionism is involved in a wide range of human behaviors and outcomes (Shafran & Mansell, 2001), the construct has garnered considerable theoretical and research attention over the years and great progress in understanding the nature, correlates, and consequences of perfectionism has been made (Bieling, Israeli, & Antony, 2004; Parker, 1997; Slade & Owens, 1998; Suh, Gnilka, & Rice, 2017). Although there is a plethora of studies about positive and negative outcomes of perfectionism (Chang, 2000; Enns, Cox, & Clara, 2005; Kanten & Yesıltas, 2015; Rice, Ashby, & Slaney, 2007; Stoeber, Hutchfield, & Wood, 2008), there are still several important questions that have yet to be adequately addressed.

Contemporary descriptions of perfectionism draw attention to the multifaceted form of the construct, where positive, as opposed to negative aspects (Slade & Owens, 1998) have been distinguished by various authors. The idea that some aspects of perfectionism might be adaptive was discussed by earlier theorists (e.g., Hamachek, 1978; Parker, 1997; Terry-Short, Owens, Slade, & Dewey, 1995). Hamachek (1978) distinguished normal from neurotic perfectionism, describing normal perfectionism as striving to meet one’s goals and excellence. Others have categorized functional as opposed to dysfunctional, healthy as opposed to unhealthy perfectionism (Parker, 1997; Terry-Short et al., 1995). In general, based on the related consequences, two different facets of perfectionism—adaptive and maladaptive—can be distinguished.

In order to investigate the conceptual and pragmatic issues dealing with two facets of perfectionism, theorists and researchers (Hamachek, 1978; Parker, 1997; Terry-Short et al., 1995) used different measures to assess their unique factors. Thus, Hill et al. (2004) found a much more efficient way and developed a new model that combined the unique aspects of each model. According to this model the perfectionism facets are merged in two higher-order domains—conscientious perfectionism (CP), including organization, planfulness, striving for excellence, and high standards for others—and self-evaluative perfectionism (SEP), which includes rumination, need for approval, concern over mistakes, and parental pressure (Hill et al., 2004). Authors address conscientious perfectionism as an adaptive and self-evaluative perfectionism as a maladaptive facet of the construct.

There has been a focus on the negative correlates and consequences of perfectionism, and much less is known about the relation of perfectionism with indicators of normal functioning. Little is known about whether and how perfectionism relates to indices of normal characteristics of personality and psychological well-being (PWB; Ryff, 1989).

Psychological functioning diverges for different classes of perfectionism. For example, various forms of adaptive perfectionism have been associated with positive factors and outcomes, such as presence of meaning, subjective happiness, and life satisfaction (Suh et al., 2017), self-efficacy and aspiration level (Stoeber et al., 2008), challenge appraisals and active coping (Stoeber & Rennert, 2008), and negatively with psychopathology (Bieling et al., 2004).

Conversely, maladaptive perfectionists exhibit less positive affect (Dunkley, Zuroff, & Blankstein, 2003), higher stress (Ashby & Gnilka, 2017; Chang, Watkins, & Banks, 2004), low academic self-efficacy, ultimately triggering academic burnout (Yu, Chae, & Chang, 2016), emotion dysregulation (Zeifman, Antony, & Kuo, 2020) and low self-compassion (Stoeber, Lalova, & Lumley, 2019).

The review of the differences between the two dimensions shows that adaptive perfectionism, namely, the perfectionistic strivings dimension, relates to positive characteristics. Conversely, maladaptive perfectionism, namely the perfectionistic concerns dimension, relates to negative psychological functioning, notably depression and anxiety (Stoeber & Otto, 2006).

The names of conscientious and self-evaluative components of perfectionism (Hill et al., 2004) were derived from the five-factor model of personality (McCrae & John, 1992), because of their conceptual similarity to conscientiousness and neuroticism.

In McCrae and Costa’s (1999) five-factor theory of personality, broad traits play a role in the development of lower-level personality characteristics. Thus, Stoeber, Otto, and Dalbert (2009) stress the notion that personality traits, in particular conscientiousness and neuroticism, not only act as mere correlates but may also be the fundament in forming the positive and negative types of perfectionism.

Several studies explored the relationship between personality traits and perfectionism facets. In most studies, the positive relation between conscientiousness and self-oriented perfectionism and neuroticism and socially prescribed perfectionism has been replicated, while other Big Five personality traits did not show a consistent pattern of correlations (Dunkley & Kyparissis, 2008; Enns et al., 2005; Rice et al., 2007; Stoeber et al., 2009).

## Perfectionism and Psychological Well-Being

Measures of psychological dysfunction, at best only moderately overlap with measures of psychological well-being, and the studies of perfectionism usually focus on dysfunction and maladaptive personality (Cheng et al., 2015; Egan, Kane, Winton, Eliot, & McEvoy, 2017; Mathew, Dunning, Coats, & Whelan, 2014); however, studies concerning the perfectionism and psychological well-being relationship are scarce.

Psychological well-being refers to positive affect states and effective social functioning (Chang, 2006; Huppert, 2009; Winefield, Gill, Taylor, & Pilkington, 2012). Ryff and Keyes (1995) argued that most conceptualizations of life satisfaction fail to provide a theory-based formulation of well-being. Ryff (1989, 1995) developed a multidimensional model of psychological well-being composed of six theoretically distinguishable functions: self–acceptance; positive relations with others; autonomy; environmental mastery; purpose in life; and personal growth.

Several studies addressing the relationship between different facets of perfectionism and well-being showed related patterns. In a study of college students (Chang, 2006) other-oriented perfectionism (adaptive) did not show relations to any of the six dimensions of psychological well-being. In contrast, socially prescribed perfectionism (maladaptive) was found to be significantly and negatively associated with all six dimensions of psychological well-being. Other studies revealed considerably similar findings. As reported, positive perfectionism affects psychological well-being positively, whereas negative perfectionism affects psychological well-being negatively (Kanten & Yesıltas, 2015). Adaptive perfectionists reported higher levels of presence of meaning, subjective happiness, and life satisfaction. Conversely, maladaptive perfectionists had higher scores in the search for meaning (Suh et al., 2017).

## Perfectionism and Flow

Since perfectionism is a personality style possibly affecting an individual’s strivings in all areas of life (Chang, 2006; Chang et al., 2015; Childs & Stoeber, 2010; Closson & Boutilier, 2017), the two forms of perfectionism also differ in terms of types of behavior.

According to the dual process model of perfectionism based on reinforcement theory, adaptive perfectionists exhibit higher achievement motivation and are optimistic about their performance and pursue success. Conversely, maladaptive perfectionists are characterized by avoidant behavior due to presumed failure and focus on mistakes rather than pride (Slade & Owens, 1998).

As perfectionism is described as striving to flawlessness and setting excessively high-performance standards (Frost, Marten, Lahart, & Rosenblate, 1990), it is considered one of the individual components of an individual’s disposition to engage in overactivity. Researchers (Childs & Stoeber, 2010; Tziner & Tanami, 2013; Zhang, Gan, & Cham, 2007) argue that positive perfectionism is positively related with work engagement. Adaptive perfectionism is related to positive emotions, thus enabling engagement in behavioral self-regulation, whereas maladaptive perfectionism is positively associated with negative emotions, self-handicapping (Shih, 2011) and procrastination (Closson & Boutilier, 2017), and negatively with academic engagement (Closson & Boutilier, 2017). Likewise, perfectionistic strivings show positive relations with behavioral, emotional, and cognitive engagement (Damian, Stoeber, Negru-Subtirica, & Băban, 2017).

Flow is the construct from positive psychology that provides a clear picture of engagement. The term was first coined by Csikszentmihalyi (1991). The experience of flow can be defined as the absorption into an activity or task at hand. The flow state derives from engaging in a challenging task and dealing with complex situations (Csikszentmihalyi, 1991).

Adaptive perfectionists regard pressure as a challenge and have a tendency to get pleasure from performance even when the task at hand requires hard work. Thus, they develop adaptive coping strategies and perform with gentle focus, without fear of evaluation. Compared to them, maladaptive perfectionists are nervous and hesitant about their performance, more prone to self-criticism and therefore tend to avoid work (Li, Lan, & Ju, 2015). Moreover, maladaptive perfectionists will exhibit a low self-awareness and immersion in an activity, which are necessary for flow state to occur (Ljubin-Golub, Rijavec, & Jurčec, 2018).

Two studies attempted to understand the relationship between flow and perfectionism from multidimensional aspects. Adaptive perfectionism dimensions (e.g., personal standards & organization) were positively associated with spiritual engagement, whereas maladaptive perfectionism dimensions (e.g., concern over mistakes, parental criticism) were negatively associated with spirituality (Chang et al., 2015). Adaptive perfectionism, namely high standards, was positively related to a higher level of academic flow (Ljubin-Golub et al., 2018). In general, the volume of research has not been matched by theoretical attempts to understand the nature of the relationship between flow and perfectionism, as most of the studies focus on academic flow. It is still not well known whether the same is true about flow as a dispositional trait, which refers to an individual’s general tendency to respond to a given activity (Csikszentmihalyi, 1991).

## Current Study

The paper reports two studies aimed at investigating the relationship between two aspects of perfectionism and psychological well-being, and the role dispositional flow plays in this relationship. Thus, in Study 1, we propose the following hypotheses: 1) Conscientious perfectionism will be positively related to psychological well-being; 2) Self-evaluative perfectionism will be negatively related to psychological well-being; 3) The relationship between conscientious perfectionism and well-being will be mediated by dispositional flow; 4) The relationship between self-evaluative perfectionism and well-being will be mediated by dispositional flow. In Study 2, we propose that 5) dispositional flow remains the mediator between perfectionism and psychological well-being after controlling for personality traits.

## Study 1

### Method

#### Participants

The study recruited a total of 156 (74 men and 82 women) participants. The majority (51%) were undergraduate students from Tbilisi State University. Participants’ ages ranged from 18 to 56 years, with a mean age of 23.29 (*SD* = 7.33) years. The participants were recruited through convenience sampling. The participants were volunteers and did not receive any reward or credit for taking part in the study.

#### Procedure

Participants completed a package of questionnaires, including measures of perfectionism, dispositional flow and psychological well-being. The questionnaires were provided in small groups and informed consent was obtained from all participants prior to completing a series of questionnaires. Participants had been assured that all data would be kept confidential.

The ethical evaluating committee of the National Foundation assessed the ethical aspects of the study.

#### Measures

##### Perfectionism Inventory (PI)

Perfectionism was measured via the Georgian version of the PI, which was originally developed by Hill et al. (2004) and is a self-report measure consisting of 59 items using a 5-point rating scale, ranging from 1 (*strongly disagree*) to 5 (*strongly agree*). The items comprise eight subscales, two higher-order components, and an overall PI composite. Acceptable reliability estimates for the eight subscales – concern over mistakes (α = .81); high standards for others (α = .63); need for approval (α = .76); organization (α = .85); perceived parental pressure (α = .82); planfulness (α = .85); rumination (α = .71); striving for excellence (α = .82) and two high-order components – conscientious perfectionism (α = .72) and self-evaluative perfectionism (α = .77) – were reported.

##### Dispositional Flow Scale-2 (DFS-2)

The Georgian version (Abuladze, 2016) of the DFS-2 (Jackson, Martin, & Eklund, 2008) comprises 36 items designed to measure an individual’s flow propensity within a given activity. It was constructed based on Csikszentmihalyi’s (1991) nine proposed components of flow, with each component assessed on a four-item scale, each item on a Likert scale ranging from 1 to 5. Participants answer the questions regarding their “experiences in general.” In the current study, coefficient alpha ranged from .62 for Merging of Action and Awareness to .87 for Clear Goals (median alpha = .78).

##### Ryff’s Psychological Well-Being Scale (PWB)

The Georgian version (Khechuashvili, 2017) of Ryff’s PWB (Ryff & Keyes, 1995) consists of 84 questions. A series of statements reflect the six areas of psychological well-being: autonomy; environmental mastery; personal growth; positive relations with others; purpose in life; and self-acceptance. Respondents rate statements on a scale of *1* to *6*, with *1* indicating *strong disagreement* and *6* indicating *strong agreement*. For each category, a high score indicates that the respondent has a mastery of that area in his or her life. Conversely, a low score shows that the respondent struggles to feel comfortable with that particular concept. In our study, the alpha internal consistency of the six scales – autonomy (α = .76); environmental mastery (α = .69); personal growth (α = .75); positive relations with others (α = .80); purpose in life (α = .85); self-acceptance (α = .80) – showed acceptable reliability.

### Data Analysis

Data analysis proceeded in three steps: 1) The initial analysis included zero-order correlations of higher-order domains of the perfectionism inventory with a global score of Dispositional Flow Scale and psychological well-being; 2) Multiple regression analysis was conducted to look for prediction value of perfectionism domains for psychological well-being and global flow; and 3) We ran mediation analysis to trace the role of flow in the perfectionism–psychological well-being relationship.

## Results

### Descriptive Statistics and Intercorrelations

All descriptive statistics are presented in Table 1.

**Table 1:** Means, Standard Deviations and Alpha Internal Consistency Coefficients

Variable	*M*	*SD*	α
Perfectionism			
CP	14.34	2.21	.72
SEP	12.91	2.42	.77
Concern Over Mistakes	2.84	0.77	.81
High Standards for Others	3.41	0.60	.63
Need for Approval	3.47	0.69	.76
Organization	3.47	0.82	.85
Perceived Parental Pressure	2.91	0.82	.82
Planfulness	3.68	0.79	.85
Rumination	3.67	0.85	.71
Striving for Excellence	3.77	0.75	.82
Flow	132.99	20.44	.93
Challenge-Skill Balance	14.85	2.74	.71
Merging of Action and Awareness	13.46	2.88	.62
Clear Goals	15.34	3.52	.87
Unambiguous Feedback	15.67	3.06	.83
Concentrating on the Task on Hand	14.82	3.38	.82
Sense of Control	14.80	3.11	.79
Loss of Self-Consciousness	13.19	3.83	.83
Transformation of Time	14.91	3.21	.74
Autotelic Experience	15.91	3.11	.82
Psychological Well-Being	350.90	47.21	.93
Autonomy	57.57	10.04	.76
Environmental Mastery	52.69	8.90	.69
Personal Growth	64.83	8.69	.75
Positive Relations with Others	59.53	10.87	.80
Purpose in Life	62.13	12.83	.85
Self-Acceptance	54.12	11.02	.80

*Note*. CP = conscientious perfectionism; SEP = self-evaluative perfectionism.

Data were tested for normality and homogeneity of variance, thereby Pearson correlation analysis was conducted. Table 2 presents the zero-order correlations for perfectionism high-order domains and scales with flow and psychological well-being. Conscientious perfectionism was positively (*r* = .23, *p* < .001) associated with psychological well-being, whilst self-evaluative perfectionism showed negative correlation with well-being (*r* = −.28, *p* < .001). Conscientious perfectionism positively moderately correlated with flow (*r* = .42, *p* < .001), whereas there was no correlation between self-evaluative perfectionism and flow. In addition, flow and psychological well-being were positively intercorrelated (*r* = .35, *p* < .001).

**Table 2:** Intercorrelations Between Perfectionism Domains, Flow and Psychological Well-Being

Variable	1	2	3
1. Conscientious Perfectionism			
2. Self-Evaluative Perfectionism	.46**		
3. Flow	.42**	.07	
4. Psychological Well-Being	.23**	−.28**	.35**

**p < .01.

### Multiple Regression

Multiple regression was used to see whether perfectionism could reliably predict either psychological well-being or flow or both. Two domains of the PI were entered simultaneously in the equation to predict global psychological well-being (PWB) and global dispositional flow (DF) total scores. Data showed that perfectionism reliably predicted both PWB and DF. Two facets of perfectionism explained the 25% variability of PWB, with both having significant effects. Perfectionism also explained the 19% of variability for DF, however, only conscientious perfectionism appeared to be the significant predictor for flow, which in turn explained 12% of well-being (Table 3).

**Table 3:** Summary of Multiple Regression Analysis for Perfectionism Factors Predicting Psychological Well-Being and Flow

Parameter	Psychological Well-Being^a^			Flow^b^		
	Β	*SE*	β	Β	*SE*	β
Conscientious Perfectionism	9.89	1.68	0.46***	4.48	0.75	0.48***
Self-Evaluative Perfectionism	−9.69	1.54	−0.49***	−1.30	0.69	−0.15

^a^*R*^2^ = .25, Δ*R*^2^ =.24 (*p* < .05). ^b^*R*^2^ = .19, Δ*R*^2^ =.18 (*p* < .05).

***p < .001.

### Mediation Analysis

**Figure 1:**
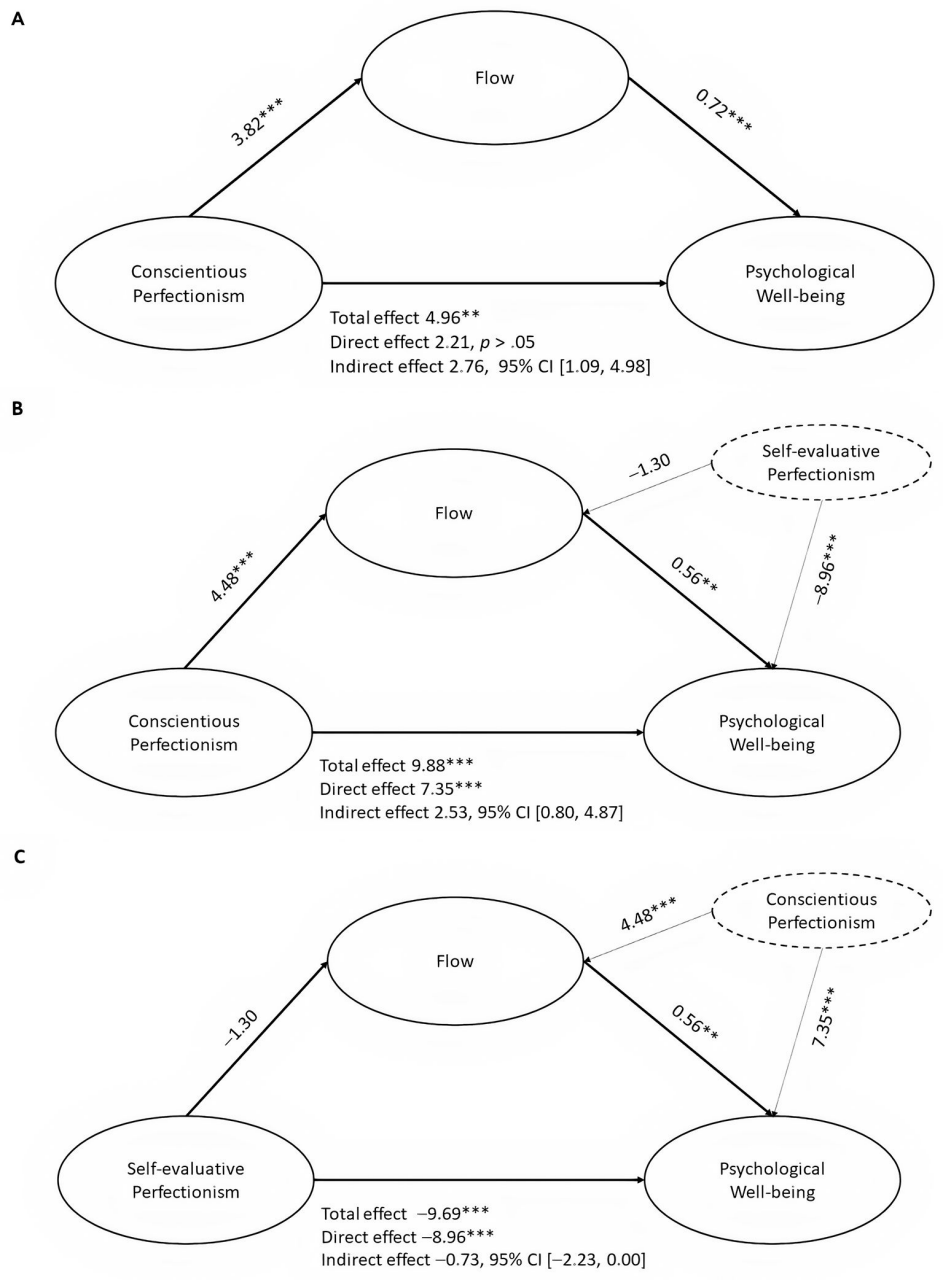
The Standardized Regression Coefficients Between Conscientious and Self-Evaluative Perfectionism and Psychological Well-Being as Mediated by Flow **p < .01. ***p < .001.

To test the mediator role of flow in the relation between perfectionism facets and well-being, we conducted mediation analysis using PROCESS (Hayes, 2017). First, we explored the model of conscientious perfectionism and psychological well-being with flow as a mediator. The proposed mediation model was tested as depicted in Figure 1A. The total score of CP had a predictive value for the flow score (*b* = 3.82, *p* < .001). The total score of flow (*b* = 0.72, *p* < .001) and CP (*b* = 4.96, *p* < .001) had a predicting value for PWB. Finally, CP had an indirect effect on PWB as dispositional flow plays the role of mediator between these two components (*b* = 2.76, 95% CI [1.09, 4.98]). The full mediating model was confirmed. Second, we explored the focal effect of CP on PWB through the mediational effect of flow by controlling SEP. As depicted in Figure 1B, CP had an indirect effect on PWB with dispositional flow partially mediating the relationship while controlling for SEP (*b* = 2.53, 95% CI [0.80, 4.87]). Finally, we tested the focal effect of SEP on PWB through the meditational effect of flow by controlling CP. The proposed model is depicted in Figure 1C. The total score of SEP did not have predictive value for the flow score (*b* = −1.30, *p* = .061). The total score of flow (*b* = 0.56, *p* = .001) and SEP (*b* = −9.69, *p* < .001) had predicting value for PWB. Finally, SEP had an indirect effect on PWB as dispositional flow plays the role of mediator between these two components (*b* = −0.73, 95% CI [−2.23, −0.00]).

## Study 2

The results of Study 1 showed that conscientious perfectionism is positively related to psychological well-being and flow plays a mediator role in between, while self-evaluative perfectionism was negatively related to psychological well-being with no significant relations with dispositional flow.

The purpose of Study 2 was to replicate and improve on our first conceptual model by means of controlling for personality traits. There are numerous studies that have addressed the issue by using the five-factor model of personality (Dunkley & Kyparissis, 2008; Flett & Hewitt, 2006; Molnar, Sadava, Flett, & Colautti, 2012; Rice et al., 2007; Stoeber et al., 2009); however, we aimed to examine this relationship using the HEXACO model (Ashton & Lee, 2007), which comprises six broad personality dimensions: Honesty-humility (H), emotionality (E), extraversion (X), agreeableness (A), conscientiousness (C), and openness (O).

### Method

#### Participants

The study used a convenience sampling technique for recruiting participants. The sample consisted of 169 (42 men and 127 women) university students. Ages ranged from 17 to 60 years, with a mean age of 24.7 (*SD* = 7.3) years. Participation was voluntary and students received no compensation for participating in the study.

#### Procedure

Participants completed a package of questionnaires, including measures of perfectionism, dispositional flow, psychological well-being and the HEXACO model of personality. Informed consent was obtained from all participants prior to completing a series of questionnaires, and students were assured of the confidentiality of their self.

#### Measures

The Georgian versions of the Perfectionism Inventory (PI), Dispositional Flow Scale-2 (DFS-2) and Ryff’s Psychological Well-Being Scale (PWB) were used, as in Study 1.

##### HEXACO Personality Inventory – Revised Short Version (HEXACO-PI-R)

The Georgian version (Martskvishvili, Mestvirishvili, Gholijashvili, Oniani & Neubauer, 2021) of the HEXACO-PI-R (Ashton & Lee, 2009) inventory consists of 60 items with 10 items for each scale. Individual subscale scores were obtained by averaging the 10 items belonging to the particular subscale. Each item was rated using a 5-point scale, ranging from 1 (*strongly disagree*) to 5 (*strongly agree*). The scale had adequate internal consistency for six scales (see Table 4).

**Table 4:** Means, Standard Deviations and Alpha Internal Consistency Coefficients for Higher Domains

Variable	*M*	*SD*	α
Perfectionism			
CP	14.35	2.24	.73
SEP	13.04	2.31	.75
Flow	134.28	20.83	.91
PWB	352.55	51.43	.94
HEXACO			
Honesty/Humility	34.82	6.56	.71
Emotionality	32.93	6.88	.75
Extraversion	32.92	7.23	.79
Agreeableness	28.46	6.56	.73
Consciousness	34.39	6.82	.75
Openness	35.61	6.26	.68

*Note*. CP = Conscientious Perfectionism; SEP = Self-Evaluative Perfectionism; PWB = Psychological Well-Being.

### Data Analysis

Data analysis proceeded in three steps: 1) The initial analysis included zero-order correlations of HEXACO scales with higher-order domains of the Perfectionism Inventory, global score of the Dispositional Flow Scale and Psychological Well-Being; 2) Multiple regression analysis was conducted to explore the prediction value of HEXACO factors for psychological well-being and flow; and 3) We ran a mediation analysis to trace the role of flow in the perfectionism–psychological well-being relationship while controlling for relevant personality traits from the HEXACO model.

## Results

All descriptive statistics are presented in Table 4.

### Correlational Analysis

Data were tested for normality and homogeneity of variance and Pearson correlation analysis was conducted. As shown in Table 5, conscientious perfectionism was positively associated with extraversion and consciousness, whereas self-evaluative perfectionism was positively related to emotionality. Flow positively related with most of the HEXACO factors with the exception of emotionality and honesty/humility. Psychological well-being was positively associated with three of the personality factors – extraversion, consciousness and openness.

**Table 5:** Intercorrelations Between HEXACO, Perfectionism, Flow and Well-Being Scales

Variable	1	2	3	4	5	6	7	8	9
1. Conscientious Perfectionism									
2. Self-Evaluative Perfectionism	.44**								
3. Flow	.46**	.04							
4. Well-Being	.25**	−.31**	.44**						
5. Honesty/Humility	−.16*	−.16*	−.17*	.14					
6. Emotionality	.05	.29**	−.14	−.11	.13				
7. Extraversion	.34**	−.07	.49**	.57**	−.17*	−.11			
8. Agreeableness	−.05	−.02	.15*	.02	.14	.13	.05		
9. Consciousness	.60**	−.05	.41**	.44**	.00	−.05	.37**	.03	
10. Openness	−.04	−.09	.18*	.27**	.17*	−.21**	.17*	−.12	.11

*p < .05. **p < .01.

### Regression Analysis

In order to determine the contributions of the HEXACO factors to components of flow and well-being, each of the six subscales of HEXACO-PI-R was regressed onto global flow and well-being, with all domains entered simultaneously (see Table 6).

**Table 6:** Summary of Multiple Regression Analysis for Perfectionism Factors Predicting Psychological Well-Being and Flow

Parameter	Psychological Well-Being^a^			Flow^b^		
	Β	*SE*	β	Β	*SE*	β
Honesty/Humility	1.76	0.48	0.22***	−0.48	0.21	−0.15*
Emotionality	−0.38	0.45	−0.05	−0.21	0.19	−0.07
Extraversion	3.54	0.46	0.49***	0.94	0.20	0.32***
Agreeableness	−0.16	0.46	−0.02	0.55	0.20	0.17**
Consciousness	1.82	0.47	0.24***	0.81	0.20	0.26***
Openness	0.88	0.51	0.10	0.42	0.22	0.12
		

^a^*R*^2^ = .46, Δ*R*^2^ = .44 (*p* < .05). ^b^*R*^2^ = .36, Δ*R*^2^ = .33 (*p* < .05).

*p < .05. **p < .01. ***p < .001.

Four of the HEXACO factors, honesty/humility (β = −0.154, *p* < .05), extraversion (β = 0.32, *p* < .001), agreeableness (β = 0.17, *p* < .01) and consciousness (β = 0.26, *p* < .001) were significant predictors and in total explained 34% of the variance of flow. In addition, the three HEXACO factors explained 44% of the variance, *R*^2^_ADJ_ = .44, *F*(6, 162) = 23.11, *p* < .001, of well-being; namely, extraversion (β = 0.49, *p* < .001), consciousness (β = 0.24, *p* < .001) and humility (β = 0.22, *p* < .001) were significant predictors (see Table 6).

### Mediation Analysis

**Figure 2:**
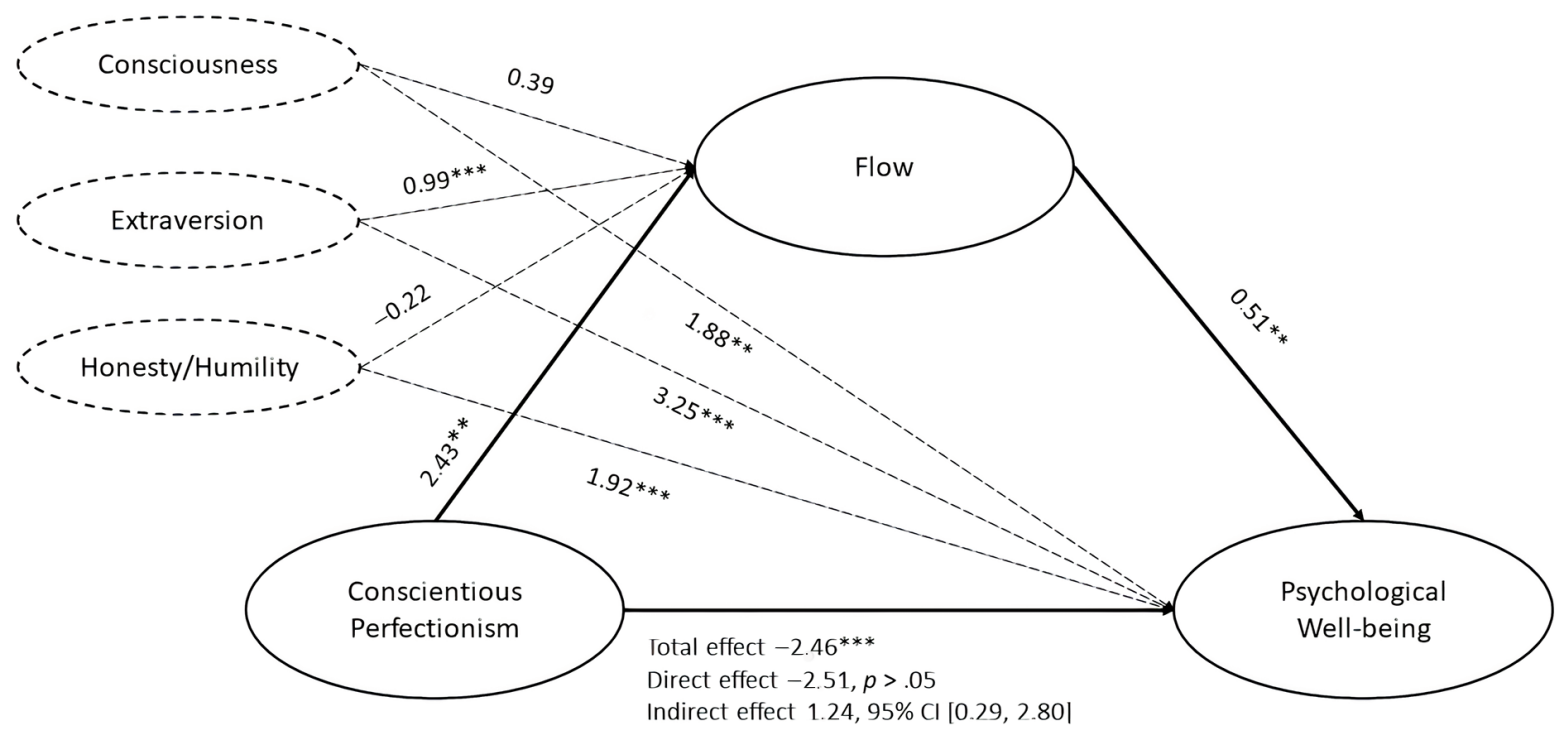
The Standardized Regression Coefficients Between Conscientious Perfectionism and Psychological Well-Being as Mediated by Flow Controlling for Conscientiousness and Extraversion **p < .01. ***p < .001.

Considering the fact that only the three HEXACO domains (Extraversion, Consciousness and Honesty/Humility) were significantly related to all study variables, we decided to include only those three in further analysis. We controlled for extraversion, consciousness and honesty/humility in the given relationship in order to exclude their potential effect on it. The proposed mediation model (Hayes, 2017) was tested as depicted in Figure 2. The relationship between CP and PWB was fully mediated by flow while controlling for relevant HEXACO domains.

## Discussion

Perfectionism is considered as a personality trait with two different facets, distinguishing adaptive versus maladaptive dimensions. Therefore, whether it is adaptive or maladaptive may have potential positive and negative effects on various psychological factors. The aim of the study was to examine the effects of conscientious and self-evaluative types of perfectionism on psychological well-being with a specific focus on dispositional flow as a mediator.

In Study 1, first we examined the relationship between different facets of perfectionism and psychological well-being to test the hypothesis that conscientious perfectionism, as a form of adaptive perfectionism would be positively associated with well-being, whereas self-evaluative perfectionism, as a form of maladaptive perfectionism would be negatively related to PWB. As hypothesized, we found general support for the notion that the adaptive dimension of perfectionism is positively associated with psychological well-being and serves as a significant predictor. While on the contrary, maladaptive perfectionism significantly predicts a lower degree of PWB. These findings are consistent with previous studies (Dunkley et al., 2003; Stoeber & Rambow, 2007; Suh et al., 2017; Yu et al., 2016). An important strength of the present study was demonstrating this relationship in the perspective of a conceptual model that differentiates conscientious versus self-evaluative perfectionism and, in fact, comprises all existing models of multifaceted perfectionism.

As the mechanism underlying the adaptive/maladaptive perfectionism and psychological well-being link is largely speculative, we posited that this relationship can be fully mediated by a psychological factor that implies the tendency to be involved in an activity in a highly enjoyable and effortless way – flow. Flow is associated with a number of positive psychological outcomes (Asakawa, 2010; Jackson, Thomas, Marsh, & Smethurst, 2001). Thus, we hypothesized that flow will be the mediator in the relationship between perfectionism facets and psychological well-being. To do so, first we explored the perfectionist’s experiences of flow. Although some studies have found a link between different facets of perfectionism and engagement (Childs & Stoeber, 2010; Damian et al., 2017; Tziner & Tanami, 2013; Zhang et al., 2007), little is known about whether and how perfectionism relates to indices of dispositional flow. To support the notion that conscientious perfectionists will more likely involve themselves in activities evoking flow experience because of their cautious attitude towards challenge and without fear of evaluation, whereas self-evaluative perfectionists will be more likely to demonstrate hesitancy facing the challenge and hence the lower degree of flow, we hypothesized that conscientious perfectionism will positively, and self-evaluative perfectionism will negatively relate to flow. We found partial support of our assumption. The positive relationship between conscientious perfectionism and flow was fully supported, with a CP as a significant predictor of dispositional flow. This finding indicates that people with high levels of conscientious perfectionism tend to try harder to achieve the perfection and thus engage in activities entirely with persistent focus and without feeling tension. Opposite to our hypothesis, there was no evidence of either positive or negative associations between self-evaluative perfectionism and flow. This suggests that non-adaptive perfectionism may not always have negative concomitants and does not necessarily mean a deadlock for autotelic experience. That is a promising avenue of investigation for the value of the heterogeneity of non-adaptive component of perfectionism.

Based on previous findings from Study 1, we tested the flow mediator role for the relationship between conscientious perfectionism and well-being. We found evidence for our hypothesis that dispositional flow affects the link between CP and PWB by playing a mediator role. To determine the focal effect of perfectionism facets on PWB, we tested the mediation analysis again, controlling for SEP. It appeared, that flow remained a mediator in the relationship between CP and PWB and SEP and PWB, but this time the mediational effect was partial. In other words, perfectionists who score high on the conscientious dimension can increase their general level of psychological well-being by immersing in activities that help them achieve high standards. Our finding is the first to highlight the importance of flow experience in understanding the perfectionism and well-being relationship.

Further, in Study 2, we went on to replicate findings from Study 1 to investigate whether these findings remain unaffected while controlling for personality traits. Due to the correlational and regression analysis, three out of six personality factors from the HEXACO model of personality caught our attention. conscientiousness, extraversion and honesty/humility were then controlled during the mediation analysis, which again incorporated flow as a mediator between conscientious perfectionism and psychological well-being. Our suggestion that the dispositional flow remains a mediator even after controlling for relevant personality traits was confirmed. This means that flow represents a powerful contributor to this relationship regardless of the heterogeneity of personality. What was more surprising, the direct relationship between conscientious perfectionism and psychological well-being became negative after controlling for conscientiousness and extraversion. For conscientiousness this can be explained by somehow overlapping the nature of the trait and conscientious dimension of perfectionism, but other studies with different models of perfectionism also show similar findings (e.g., Stoeber et al., 2009). The impact of honesty/humility on the perfectionism and well-being relationship may be explained by disinterest in achieving elevated excellence (Ashton, Lee, & de Vries, 2014). As for Extraversion, existing empirical findings are uncertain (Dunkley & Kyparissis, 2008; Flett & Hewitt, 2006; Molnar et al., 2012; Rice et al., 2007; Stoeber et al., 2009). A possible explanation may be the factors related to extraversion, such as assertiveness and activity (Stoeber, Corr, Smith, & Saklofske, 2018). The latter is worth discussing as it challenges the view of a positive association between adaptive perfectionism and well-being.

### Limitations and Future Directions

Several potential limitations to the present study must be mentioned. First, due to the limited number of participants, the study did not investigate the complex relationships between construct subscales, which could provide new insights. It would be useful for future research to further examine the flow and perfectionism by employing larger sample size, different sample group and specific aspects of perfectionism. Second, our study involved a cross-sectional design, therefore strong inferences about causal and mediational effect from the findings cannot be drawn. Third, perfectionism is a gender-specific variable and the effect of gender may have altered the results, however there was no gender difference in our study, thus preventing us from possible effect of it. Further studies may focus on gender differences. Last, we only examined one aspect of normal functioning. Future studies could explore more specific positive outcomes of the flow and perfectionism relationship.

Nevertheless, the presented work enriches the extant literature in several important ways. First, the study highlights the importance of exploring the distinct aspects of perfectionism separately. Second, it represents the first demonstration of the link between perfectionism and psychological well-being by providing insight into the underlying mechanisms and highlighting the power of personality characteristics that contributes to understanding the relationship.
